# Neonatal BCG and Hepatitis B Vaccination and Incidence of Atopic Dermatitis in Children by 36 Month of Age: Results of Prospective Study

**DOI:** 10.3390/vaccines14040343

**Published:** 2026-04-14

**Authors:** Leyla Namazova-Baranova, Natalya Klimova, Marina Fedoseenko, Dina Rusinova, Vera Merkulova, Elina Bulatukova, Pavel Levin, Polina Polikhova, Aleksandra Korchagina

**Affiliations:** 1 Pirogov Russian National Research Medical University (Pirogov University), Moscow 117513, Russiaelina_bulatukova@mail.ru (E.B.);; 2 Research Institute of Pediatrics and Children’s Health, Petrovsky National Research Centre of Surgery, Moscow 119991, Russia; 3 Shenzhen MSU-BIT University, Shenzhen 518172, China; 4 Children’s City Outpatient Clinic No. 110, Moscow 127490, Russia; 5 Children’s City Outpatient Clinic No. 133, Moscow 125445, Russia; 6 Dmitry Rogachev National Medical Research Center of Pediatric Hematology, Oncology and Immunology, Moscow 117997, Russia

**Keywords:** vaccination, children, BCG, atopic dermatitis

## Abstract

Background: The steady increase in allergic diseases among children has coincided with increased global vaccination coverage and the expansion of routine childhood immunization programs. This has contributed to the widespread belief that there is a possible link between immunoprophylaxis and allergic diseases. However, a number of scientific studies have demonstrated the protective effect of early neonatal immunization on the development of nonspecific immunological protection against infections. This is believed to be due to a shift in the immune response from the Th2 type, traditionally predominant in newborns, to the Th1 type, which reduces the risk of developing allergic diseases. Methods: This prospective cohort study analyzed the medical records of 2279 children born between 2018 and 2022 to evaluate the impact of neonatal BCG-M and hepatitis B (HepB) vaccination on the incidence of atopic dermatitis (AD) by 36 months of age. Factors analyzed included family history of allergy, cesarean section, prematurity, delayed initiation of breastfeeding, maternal antibiotic use during pregnancy, and antibiotic use in the child during the first three years of life. Results: The cumulative incidence of AD by 36 months of age was 19.9%. Timely neonatal vaccination coverage was 76.2% for BCG-M and 69.2% for HepB; by 12 months of age, these rates increased to 90.2% and 88.5%, respectively. A full-term birth demonstrated a significant protective effect (OR 0.52; 95% CI 0.30–0.93). A positive family history of allergy was the strongest predictor of AD (OR 21.49; 95% CI 14.4–32.9). Cesarean section was also significantly associated with AD (OR 1.30; 95% CI 1.01–1.65). AD incidence was comparable between vaccinated (20.5%) and non-vaccinated (17.5%) children (chi-squared with Yates’ correction, *p* = 0.192), indicating no statistically significant overall impact of immunization on AD risk. Conclusions: The development of AD is primarily driven by hereditary predisposition and specific perinatal factors rather than by routine immunization. These findings confirm that neonatal BCG-M and HepB vaccination does not increase the risk of AD, providing a scientific basis to address vaccine hesitancy.

## 1. Introduction

Over the past few decades, a steady increase in the incidence of allergic diseases has been documented in developed countries [1,2,3]. In particular, there has been a significant rise in the prevalence of atopic dermatitis (AD)—a systemic, multifactorial, genetically determined inflammatory skin disease characterized by pruritus, a chronic relapsing course, and age-dependent patterns of lesion localization and morphology [4]. According to various reports, AD affects 15–20% of children and 2–8% of adults worldwide. This trend is a cause for serious concern due to the lack of a comprehensive prevention strategy, necessitating the search for new evidence-based approaches to address this problem [1,2,4].

To date, the primary factors predisposing to the development of allergic diseases have been well established. These include a positive family history of allergy, formula feeding, and/or delay in the initiation of breastfeeding in the immediate postnatal period, as well as maternal antibiotic use during pregnancy or antibiotic administration to the child during the first years of life [5,6,7]. The impact of these and other factors continues to be actively investigated. Researchers in various countries are exploring the potential link between allergic diseases and immunization, partly due to the unsubstantiated public perception that “vaccination causes allergies.” This misconception leads to lower vaccine uptake and increased vaccine hesitancy, despite the repeatedly proven benefits and critical role of immunization in maintaining the health of both pediatric and adult populations [4,8,9,10].

The relationship between atopic diseases and neonatal immunoprophylaxis—against tuberculosis (BCG) and hepatitis B (HepB)—has been extensively investigated [1,8,9,10,11]; however, available evidence remains inconclusive. One of the earliest studies analyzing the incidence of allergic diseases in children vaccinated and unvaccinated with BCG and the whole-cell pertussis (wP) vaccine was conducted in Germany in 1996 and subsequently published in 2007. That study included 1673 children aged 5–7 years and comprehensively analyzed vaccination history, dermatological examination findings, and skin prick test results. The findings revealed no association between immunization and an increased risk of asthma, AD, or allergic rhinitis [12]. Subsequent studies also failed to demonstrate that vaccination increases the incidence of atopic diseases [1,13,14,15]. A meta-analysis of data from 20 studies, published in 2021, showed that BCG vaccination in early childhood does not significantly influence the risk of AD but is associated with a reduced risk of bronchial asthma [1]. Another systematic review and meta-analysis from the same year, which included data from 35 cohort studies and seven randomized clinical trials, identified a statistically significant link between early neonatal tuberculosis vaccination and a reduced risk of AD, suggesting a potential protective effect [9].

However, in recent years, a growing body of evidence has suggested that neonatal BCG vaccination may confer a protective effect against the development of allergic diseases [8,9,16]. It is well established that the pathogenesis of AD is dominated by a Th2-type immune response with subsequent hyperproduction of total IgE [17]. Clinical studies have shown that BCG immunization in the first hours and days of life—a period characterized by physiological predominance of the Th2 response—promotes a shift from a Th2-type to a Th1-type immune response. This transition, in turn, may reduce the risk of allergic diseases manifesting in the first years of life [1]. Furthermore, the BCG vaccine has been found to induce non-specific activation of innate immune cells, a phenomenon known as “trained immunity”, leading to increased pro-inflammatory activity and altered cytokine synthesis [18,19,20]. This immunomodulatory effect facilitates a more balanced Th1/Th2 profile in early infancy, which may serve as a primary preventive mechanism against the development of atopic dermatitis [1,18,19,20].

Furthermore, BCG is recognized for its heterologous clinical benefits beyond tuberculosis prevention. Early neonatal administration of the BCG vaccine has been associated with a significant reduction in overall infant mortality, an effect primarily attributed to a lower incidence of neonatal sepsis, acute respiratory infections, and febrile syndromes [21,22,23]. Recent immunological research has established that these non-specific advantages stem from the epigenetic and metabolic reprogramming of innate immune cells, particularly monocytes (myeloid lineage) and natural killer (NK) cells. This phenomenon, known as “trained immunity”, results in an enhanced and more robust antimicrobial response [24]. Importantly, recent studies have shown that hepatitis B vaccination not only induces adaptive immune memory but also activates NK cell responses [25]. This suggests that innate immune memory mechanisms can also be induced by non-live vaccines. There is also evidence that recombinant hepatitis B vaccination does not increase the risk of allergic diseases [26]. Additionally, evidence also indicates that BCG provides a degree of cross-protection not only against various bacterial and fungal pathogens but also against viral agents, including influenza and SARS-CoV-2 [27,28,29]. Specifically, large-scale ecological studies encompassing 178 countries have explored the correlation between national BCG vaccination policies and COVID-19 epidemiological data. These investigations led researchers to hypothesize a protective role for BCG, as countries maintaining universal routine immunization against tuberculosis demonstrated notably lower morbidity and mortality rates associated with SARS-CoV-2 [19,30].

Based on the evidence described above, a clinical study was designed to investigate the relationship between neonatal vaccination and atopic diseases, taking into account various confounding factors. In 2022, we published the results of a pilot study involving 307 children followed from birth to 24 months of age, which aimed to evaluate the presence of a negative association (i.e., a potential protective effect) between neonatal vaccination against tuberculosis and hepatitis B and the development of atopic dermatitis in children [16]. However, despite the clinical significance of these findings, the sample size in the pilot study was insufficient to confirm statistical power. Therefore, based on those preliminary results, a sample size calculation was performed, and a sufficiently powered prospective study was initiated.

## 2. Materials and Methods

### 2.1. Study Design

This prospective observational cohort study included 2279 children born between January 2018 and January 2022, all enrolled at Children’s Outpatient Clinics No. 133 and No. 110 (Moscow), which share a unified electronic medical information system. This centralized digital infrastructure provided access to comprehensive real-world data, ensuring longitudinal tracking of each participant.

Participant recruitment began in January 2018 and continued until January 2022. Follow-up ended in January 2025. These timings ensured that all children included in the final analysis were 36 months of age. Children were included in the group as they were assigned to a clinic after being discharged from the maternity hospital.

According to current Russian legislation, all infants are entitled to state-funded preventive examinations by a pediatrician and specialty physicians. These examinations are conducted according to a standardized schedule (presented in Table 1) and are designed to monitor growth, psychomotor development, nutritional status, general health, and immunization. The children in our study underwent routine preventive checkups at a municipal outpatient clinic at least once every three months.

The primary goal of our study was to investigate whether neonatal vaccination plays a role in the development of AD, as it is one of the most common clinical forms of allergic diseases. At the same time, to obtain the most valid results, we believe it is necessary to take into consideration the factors predisposing to the early onset of AD.

Vaccination status was extracted from neonatal discharge summaries and categorized based on compliance with the Russian National Immunization Schedule (Orders No. 1122n and 125n) [31]. The study evaluated the following protocols: BCG-M: A live attenuated vaccine (Mycobacterium bovis BCG-1 Russian substrain) with reduced antigen content, administered intradermally. Timely vaccination was defined as administration between days 3 and 7 of life; any dose given after day 7 was recorded as a delay. Hepatitis B (HepB): A recombinant subunit vaccine administered intramuscularly. Timely vaccination (birth dose) was defined as the first dose administered within the first 24 h postpartum, followed by the standard 0–1–6 month schedule.

### 2.2. Data Collection and Variables

Medical records were reviewed prospectively after each scheduled follow-up visit at 3, 6, 9, 12, 18, 24, and 36 months of age. Data were extracted from the Unified Medical Information and Analytical System (EMIAS) of Moscow. For each participant, the following variables were assessed at each time point:

- Vaccination Record Card (Form No. 063/u);
- Child Development History (Form No. 112/u);
- Maternal Exchange Card (pregnancy and neonatal record, Form No. 113/u-20);
- Neonatal discharge summaries.

Of 2648 screened records, 2279 met the eligibility criteria and were included in the final analysis using consecutive sampling.

For each participant, the following variables were assessed:

- Vaccination status: Timely vs. delayed immunization against tuberculosis (BCG-M) and hepatitis B.
- Predisposing risk factors: Family history of allergy, maternal antibiotic use during pregnancy, gestational age (preterm/term), mode of delivery (vaginal/cesarean), timing of breastfeeding initiation, and antibiotic use in the child during the first 36 months of life.
- Clinical outcome: Physician-confirmed diagnosis of atopic dermatitis (verified by an allergist-immunologist). The diagnosis was established based on the diagnostic criteria of Hanifin and Rajka, in accordance with current Russian clinical guidelines. The diagnosis required the presence of pruritus and at least three of the following major features: typical morphology and distribution of skin lesions (facial and extensor involvement in infants), chronic relapsing course, and personal or family history of atopy [4].
- Monitoring: Health status and clinical variables were assessed at 3, 6, 9, 12, 18, 24, and 36 months of age.

### 2.3. Inclusion Criteria

To be eligible, children had to meet the following criteria:

1. Born between 2018 and 2022 and registered at Children’s Outpatient Clinics No. 133 or No. 110 (Moscow).
2. Gestational age ≥ 35 weeks.
3. No absolute contraindications to neonatal vaccination at birth.
4. Availability of a completed Maternal Exchange Card (Form No. 113/u-20).
5. Availability of a completed Vaccination Record Card (Form No. 063/u).
6. Written informed consent from parents or legal guardians for the processing of personal and medical data.

### 2.4. Exclusion Criteria

Children were excluded from the study if they were de-registered from the participating clinics during the follow-up period. Specific reasons for exclusion included relocation or transfer to other healthcare facilities.

**Figure 1:**
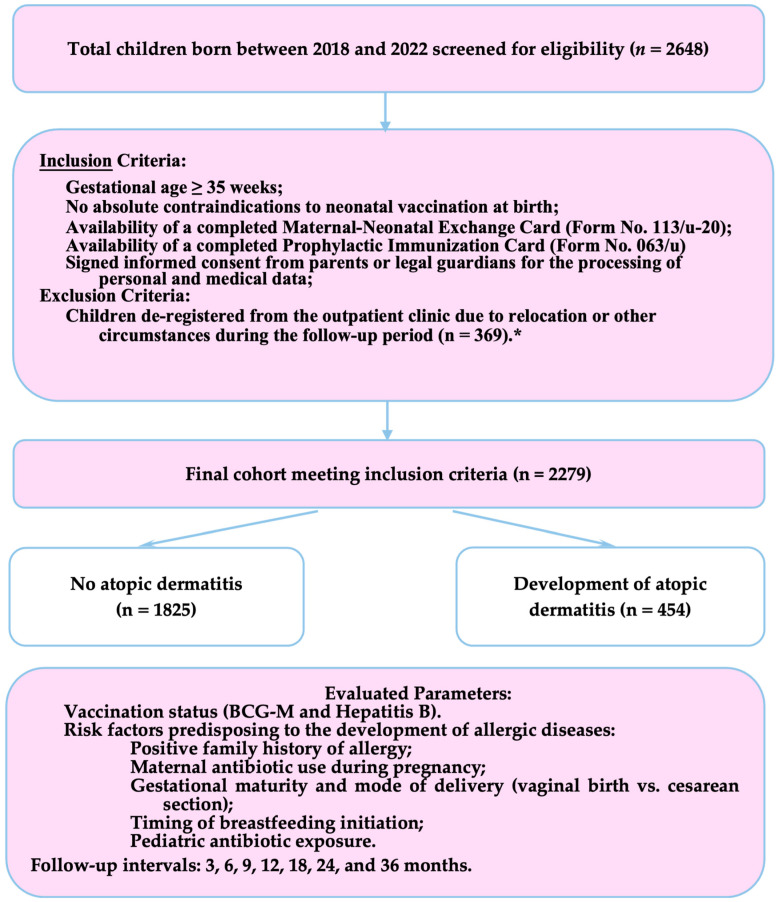
Study design and participant flow. * Exclusion criteria: Children de-registered from the outpatient clinic due to relocation (n = 48) or transfer to other healthcare clinic (n = 321) during the follow-up period. Total excluded: n = 369 (attrition rate 13.9%).

The study flowchart is presented in Figure 1.

### 2.5. Ethical Approval

This study was a prospective cohort study based on medical records. In accordance with Russian Federation legislation (Federal Law No. 152-FZ of 27 July 2006, “On Personal Data,” Article 10, Paragraph 4), written informed consent for the processing of personal data was obtained from the parents or legal guardians of all participants. As the study involved only the analysis of de-identified and anonymized data extracted from routine medical records with no direct patient interventions, formal review by an ethics committee was not required under national regulations. The study was conducted in accordance with the ethical principles of the Declaration of Helsinki.

### 2.6. Statistical Analysis

A sample size calculation was performed during the planning phase to ensure adequate statistical power to detect associations between neonatal vaccination (BCG-M and Hepatitis B) and the incidence of AD in the first three years of life.

The sample size was estimated based on preliminary findings [16] which reported significant differences in AD incidence depending on the timing of neonatal BCG-M vaccination. This study showed that in a cohort of 300 children who received both vaccines in the early neonatal period, the risk of developing AD during the first 18 months was significantly lower compared to those who were not vaccinated or vaccinated later.

For the current study, we adopted a more conservative and clinically meaningful hypothesis, estimating that neonatal vaccination would reduce AD incidence by 5 percentage points (from 20% in the unvaccinated group to 15% in the vaccinated group). This margin was selected to account for broader population heterogeneity. Additional parameters included: an estimated vaccination coverage of 76.2%; a two-sided significance level (α) of 0.05; and a target power (1 − β) of 0.85. The required sample size was determined to be 2279 participants. Calculations were performed using the pwr package in RStudio (v. 2024.12.1, Posit Software, Boston, MA, USA).

Data were collected and systematized using Microsoft Excel 2010. Statistical analysis was performed using StatTech v. 4.11.2 (Stattech LLC, Russia) and RStudio. Categorical variables are presented as absolute frequencies and percentages with 95% confidence intervals (CI). Continuous variables (e.g., age at AD onset) were compared between independent groups using the Mann–Whitney U test.

To identify factors associated with AD onset by 36 months, univariate logistic regression was employed. Results are expressed as crude odds ratios (OR) with 95% CIs. The comparison of proportions between vaccinated and non-vaccinated groups, as well as the assessment of AD incidence by vaccination status, was performed using the chi-squared test with Yates’ continuity correction. For small cell counts in antibiotic exposure sub-analyses, Fisher’s exact test was applied where appropriate. Statistical significance level was set at 0.05.

## 3. Results

### 3.1. Study Population Characteristics

A total of 2279 children born between 2018 and 2022 were included in the analysis. The cohort comprised 1061 girls (46.6%) and 1218 boys (53.4%). Vaginal delivery occurred in 1817 (79.7%) and cesarean section in 462 (20.3%). Preterm birth (gestational age < 37 weeks) was recorded in 56 children (2.5%), of whom 23 children were born by cesarean section (41.1%). Breastfeeding was initiated in the delivery room in 2086 newborns (91.5%).

Neonatal BCG-M vaccination was administered to 1736 infants (76.2%) and neonatal HepB vaccination to 1577 (69.2%). By 12 months of age, vaccination coverage increased: 2055 children (90.2%) had received at least one dose of BCG-M, and 2016 (88.5%) had received at least one dose of the hepatitis B vaccine. Among children not fully vaccinated in the neonatal period, 161 (7.1% of the total cohort) received both vaccines during the first year of life.

A positive family history of allergy was documented in 154 children (6.8%). Maternal antibiotic use during pregnancy was recorded in 108 cases (4.7%). Antibiotic use in the child during the first three years of life was documented in 944 children (41.4%), including 250 (11.0%) in the first year, 340 (14.9%) in the second year, and 633 (27.8%) in the third year.

By 36 months of age, the cumulative prevalence of atopic dermatitis (AD) was 19.9% (454 children).

Baseline characteristics of the study cohort are presented in Table 2.

### 3.2. Univariate Logistic Regression Analysis

To identify factors associated with AD by 36 months of age and to estimate the strength of these associations, univariate logistic regression was performed. This approach allows for the assessment of the crude association between each individual predictor and the outcome. The results of the regression analysis are presented in Table 3.

**Figure 2:**
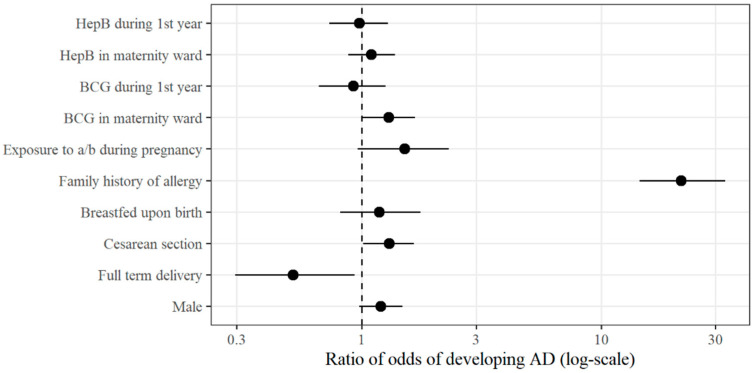
Forest plot of crude odds ratios (OR) for atopic dermatitis risk factors. **Notes:** The vertical line represents an OR of 1.0 (no effect). Points to the right of the line indicate increased risk, while points to the left indicate a protective effect. Statistical significance is achieved when the 95% CI error bars do not intersect the vertical line.

The associations between clinical/anamnestic factors and the incidence of AD are visualized in Figure 2. Factors such as family history of allergy (OR 21.49), neonatal BCG-M vaccination (OR 1.29), and cesarean section (OR 1.30) showed a statistically significant positive association with AD, as their 95% CI did not cross the line of null effect (OR = 1). Conversely, full-term birth (OR 0.52) demonstrated a significant protective effect. Univariate analysis identified these factors as the primary predictors of AD onset by 36 months of age.

### 3.3. Comparison of AD Incidence by Neonatal Vaccination Status

To further evaluate the relationship between neonatal immunization and the development of atopic dermatitis, a detailed comparison of proportions was conducted. The analysis distinguished between vaccination in the maternity hospital (within the first days of life) and vaccination by 12 months of age (allowing for delayed administration) with BCG-M and hepatitis B vaccines. Table 4 presents the results of these comparisons.

The cumulative incidence of AD by 36 months of age was 20.8% in the vaccinated group versus 18.4% in the non-vaccinated group for vaccination in the maternity hospital (*p* = 0.175), and 20.6% versus 16.7% for vaccination by 12 months of age (*p* = 0.246). BCG-M and hepatitis B vaccination status showed no association with either early manifestation of AD or its development at the age of 2–3 years.

These findings indicate that neonatal vaccination, whether defined as receipt of at least one vaccine or both BCG-M and hepatitis B vaccines, is not associated with developing atopic dermatitis by 36 months of age. The overall pattern of results supports the conclusion that routine immunization in the neonatal period does not adversely affect the risk of atopic dermatitis in early childhood. Additionally, children who received at least one vaccine in the maternity hospital had a cumulative AD incidence of 20.5% (382/1867) compared to 17.5% (72/412) among those who received no vaccine, with no statistically significant difference (*p* = 0.192).

### 3.4. Association Between Antibiotic Exposure and AD Onset

To evaluate the potential impact of early-life antibiotic (AB) use on the development of atopic dermatitis, we analyzed the association between AB exposure in specific time windows and the subsequent onset of AD (Table 5).

Among children exposed to antibiotics during the first year of life, the incidence of AD onset between 12 and 24 months of age was 6.4% (16/250), compared to 4.7% (96/2029) in the non-exposed group. This difference did not reach statistical significance (OR 1.377; 95% CI 0.797–2.378).

Antibiotic exposure during the second year of life showed no significant association with AD onset between 24 and 36 months of age. In this period, the incidence of AD was 0.9% (3/340) in the AB-exposed group versus 2.0% (39/1939) in the non-exposed group ((OR 0.434; 95% CI 0.133–1.411).

These findings indicate that systemic antibiotic therapy during the first two years of life is not a significant predictor of atopic dermatitis onset by the age of 36 months in the studied cohort.

## 4. Discussion

This study identified a complex set of factors associated with the development of atopic dermatitis (AD) by 36 months of age. The analysis confirmed the dominant role of genetic predisposition and identified several independent perinatal and postnatal factors that are key contributors to the development of AD. No statistically significant association was found between neonatal vaccination and the risk of developing AD in the recruited cohort. These results support the conclusion that routine immunization schedules do not increase the cumulative incidence of atopic dermatitis during the first three years of life.

### 4.1. Vaccination

In univariate logistic regression, neonatal BCG-M vaccination showed a statistically significant association with AD (OR 1.29; 95% CI 1.01–1.67). However, when comparing AD incidence using the chi-squared test with Yates’ continuity correction, no statistically significant differences were observed across any vaccination strategy or age period.

For children vaccinated with both BCG-M and hepatitis B vaccines in the maternity hospital, the cumulative incidence of AD by 36 months was 20.8% compared to 18.4% in the non-vaccinated group (*p* = 0.175). When both vaccines were administered by 12 months of age, the corresponding incidence was 20.6% versus 16.7% (*p* = 0.246). The same way, children who received at least one vaccine in the maternity hospital had an AD incidence of 20.5% versus 17.5% among those who received no vaccine (*p* = 0.192). A borderline effect was noted for AD onset at 12–24 months among children vaccinated with both vaccines in the maternity hospital (5.6% vs. 3.7%, *p* = 0.058), but this did not reach conventional statistical significance.

These findings suggest that while univariate analysis identified a modest association for BCG-M, the overall pattern of results does not support a clinically meaningful increase in AD risk attributable to neonatal immunization. These findings are consistent with the majority of contemporary studies [1,2,9,12] and refute the hypothesis that routine immunization increases the risk of allergic disease [32]. While some reports have suggested a potential protective effect [15,32,33], our current results did not reach statistical significance, indicating that this question requires further investigation.

Neonatal vaccination coverage was in this cohort 76.2% for BCG-M and 69.2% for hepatitis B. These figures are consistent with our previously published data [16]. By 12 months, immunization coverage in our cohort approached national averages for the Russian Federation [34]: 90.2% for BCG-M and 88.5% for hepatitis B.

### 4.2. Demographic and Perinatal Factors

In univariate analysis, full-term birth was associated with a significantly lower odds of AD (OR 0.52; 95% CI 0.30–0.93), corresponding to an approximately 1.9-fold higher odds for preterm infants, although the latter was not directly assessed due to the small number of preterm children (n = 56, 2.5%) in the cohort. This finding diverges from several studies reporting a lower incidence and later onset of AD in preterm infants [35,36]. The protective effect described elsewhere has been attributed to early postnatal microbial exposure in the case of passage through the natural birth canal and breastfeeding immediately after birth, which may promote immune tolerance and enhance Th1-type responses [35,36], as well as to premature cessation of intrauterine Th2-type cytokine exposure (e.g., IL-4, IL-13) [37]. The discrepancy observed in our study likely reflects the specific inclusion criteria (gestational age ≥ 35 weeks), which excluded very preterm infants and thus limited the analysis of this particular subgroup.

Cesarean section was associated with increased odds of AD in univariate analysis (OR 1.30; 95% CI 1.01–1.65). This finding is consistent with large cohort studies, including a Swedish national study of over 1.3 million children (aOR = 1.12; 95% CI: 1.10–1.14) [38] and the prospective PreventADALL cohort (aOR = 1.33; 95% CI: 1.02–1.74) [39]. The leading hypothesis involves disrupted maternal–infant microbial transmission, as vaginal delivery facilitates neonatal gut and skin colonization with maternal microbiota—a critical step for immune system priming [38]. Nevertheless, evidence remains conflicting. The Japanese JECS study (n = 74,639) found no association between CS and AD in the first year of life [40], and a large US cohort study reported an attenuation of the association after confounder adjustment [41].

Child sex was not independently associated with AD in our multivariate model. However, the univariate trend toward higher AD incidence in male infants is consistent with studies attributing this to genetic factors and sex-specific differences in skin barrier development [42,43,44]. Our findings do not contradict studies reporting higher AD prevalence in girls [45], as the peak incidence in females typically occurs at age 5–9 years, which was beyond the observation follow-up period of this study.

### 4.3. Family History of Allergy

A positive family history of allergy was the strongest predictor of AD in our cohort, with a 21.5-fold increase in adjusted odds. This striking association underscores the major role of genetic predisposition in AD pathogenesis and aligns with large contemporary studies [4,7,44,46]. In a meta-analysis by Ravn et al., parental history of allergy conferred a 1.8-fold increased risk, with the highest estimates observed when the family history was specific to AD [7]. Similarly, a 2024 meta-analysis by Ma et al. reported 2.7-fold increased odds of AD among Chinese children aged 1–7 years with a family history of allergy [46].

The substantially higher OR observed in our study likely reflects more than a purely genetic effect; it suggests a synergistic interaction between heredity and other modifiable risk factors (such as antibiotic exposure and cesarean section). This highlights that in clinical practice, children at greatest risk are those presenting a genetic predisposition combined with additional unfavorable environmental or epigenetic risk factors.

### 4.4. Antibiotic Exposure

In our study, no statistically significant association was found between antibiotic use during the first years of life and the risk of AD onset by 36 months.

Among children who received antibiotics during the first year of life, the incidence of AD onset at 12–24 months was 6.4% compared to 4.7% in the non-exposed group. This difference did not reach statistical significance ( OR 1.377; 95% CI 0.797–2.378).

Antibiotic exposure during the second year of life showed no significant association with AD onset at 24–36 months. The incidence of AD in this period was 0.9% in the AB-exposed group versus 2.0% in the non-exposed group (OR 0.434; 95% CI 0.133–1.411).

These findings diverge from a large systematic review and meta-analysis of 160 observational studies, which reported a pooled OR of 1.40 (95% CI: 1.30–1.52) for the association between childhood antibiotic exposure and AD [47]. The lack of significance in our cohort may be attributed to the specific timing of exposure or the relatively high baseline of antibiotic use across the study population. While the proposed mechanism often involves antibiotic-induced gut dysbiosis and a Th2-skewed immune response [6,47], our data suggest that in this specific clinical setting, antibiotic exposure might not be a primary driver of AD development independently of other factors.

Maternal antibiotic use during pregnancy also showed a trend toward increased AD risk in our univariate analysis, but this association did not reach statistical significance. This finding remains inconclusive: while it is partially consistent with some studies reporting an association between prenatal antibiotic exposure and the atopic march [10], it also reflects the substantial heterogeneity and limited evidence currently available in this area [48].

### 4.5. Limitations

Several limitations should be acknowledged in this study. First, the accuracy of data recorded in the original medical records could not be independently verified. Incomplete documentation may have introduced potential inaccuracies, particularly regarding the number and timing of antibiotic courses, as such information is not always fully reported to physicians or captured in electronic records.

Second, the possibility of unmeasured confounding cannot be excluded. Potential unaccounted risk factors for AD not included in our analysis include socioeconomic status, dietary patterns, secondhand smoke exposure, pet ownership, and other environmental influences.

Third, the inclusion criterion of gestational age ≥35 weeks restricted the analysis of moderate-to-severe prematurity. Our findings regarding preterm birth as a risk factor may not be generalizable to infants born at earlier gestational ages.

## 5. Conclusions

This prospective cohort study demonstrates that vaccination with BCG-M and hepatitis B vaccines, whether administered in the maternity hospital or during the first year of life, is not associated with an increased risk of developing atopic dermatitis (AD) by three years of age. The strongest predictor of AD was a positive family history of allergy (OR 21.49; 95% CI 14.4–32.9). Neither neonatal BCG and hepatitis B vaccination nor vaccination administered during the first year of life was associated with an increased risk of early AD manifestation. Pediatric antibiotic use during the first three years of life did not show a statistically significant association with AD onset in this study. Thus, our findings provide evidence that routine neonatal immunization does not increase the risk of AD, which may serve as a scientific basis for addressing vaccine hesitancy stemming from unsubstantiated concerns.

## Figures and Tables

**Table 1:** Schedule of well-child visits and checkups. “+” means the conducting of a doctor’s exam or procedure.

Age, Months	1	2	3	4	5	6	7	8	9	10	11	12	15	18	24
Pediatrician	+	+	+	+	+	+	+	+	+	+	+	+	+	+	+
Neurologist	+											+			
Ophthalmologist	+											+			
Otolaryngologist												+			
Pediatric surgeon	+											+			
Orthopedist			+									+			
Pediatric dentist	+														+
Pediatric psychiatrist															+
Complete blood count		+										+			
Urinalysis		+										+			
Abdominal ultrasound exam	+														
Hip ultrasound exam	+														
Neurosonography	+														
Echocardiography	+														
Electrocardiography												+			

**Table 2:** Baseline characteristics of the study cohort (N = 2279).

Indicators	Categories	Number (N)	Share (%)	95% CI
Sex	Girls	1061	46.6	44.5–48.6
	Boys	1218	53.4	51.4–55.5
Gestational age	Preterm	56	2.5	1.9–3.2
	Full-term	2223	97.5	96.8–98.1
Mode of delivery	Vaginal delivery	1817	79.7	78.0–81.4
	Cesarean section	462	20.3	18.6–22.0
Breastfeeding initiation in the delivery room	Not initiated	193	8.5	7.4–9.7
	Initiated	2086	91.5	90.3–92.6
Family history of allergy	Negative	2125	93.2	92.1–94.2
	Positive	154	6.8	5.8–7.9
BCG-M in the maternity hospital	Not vaccinated	543	23.8	22.1–25.6
	Vaccinated	1736	76.2	74.4–77.9
HepB in the maternity hospital	Not vaccinated	702	30.8	28.9–32.7
	Vaccinated	1577	69.2	67.3–71.1
Both BCG-M and Hepatitis B in the maternity hospital	Not vaccinated	832	36.5	34.5–38.5
	Vaccinated	1447	63.5	61.5–65.5
BCG-M by 12 months of age	Not vaccinated	224	9.8	8.6–11.1
	Vaccinated	2055	90.2	88.9–91.4
HepB by 12 months of age	Not vaccinated	263	11.5	10.3–12.9
	Vaccinated	2016	88.5	87.1–89.7
Both BCG-M and HepB by 12 months of age	Not vaccinated	180	7.9	6.8–9.1
	Vaccinated	2099	92.1	90.9–93.2
Maternal antibiotic use during pregnancy	No	2171	95.3	94.3–96.1
	Yes	108	4.7	3.9–5.7
Pediatric antibiotic exposure	No	1335	58.6	56.5–60.6
	0–12 months	250	11.0	9.7–12.3
	12–24 months	340	14.9	13.5–16.4
	24–36 months	633	27.8	26.0–29.7
	Total (0–36 months)	944	41.4	39.4–43.5
Age of Atopic Dermatitis (AD) onset	No AD until 36 months	1825	80.1	78.4–81.7
	AD onset at 0–12 months	301	13.2	11.8–14.7
	AD onset at 12–24 months	112	4.9	4.1–5.9
	AD onset at 24–36 months	41	1.8	1.3–2.4
	Total AD cases (0–36 months)	454	19.9	18.3–21.6
AD incidence by vaccination status	Vaccinated (any vaccine)	382	20.5	18.7–22.4
	Not vaccinated	72	17.5	14.0–21.5

**Notes:** BCG-M, *M. bovis* BCG-1 (Russia; live attenuated tuberculosis vaccine with reduced antigen content); HepB, hepatitis B vaccine; AD, atopic dermatitis.

**Table 3:** Univariate logistic regression analysis of risk factors associated with atopic dermatitis (N = 2279).

Characteristic	OR	95% CI
Male	1.20	0.98–1.48
Full term	0.52	0.30–0.93
Cesarean section	1.30	1.01–1.65
Breastfed upon delivery	1.18	0.81–1.76
Family history of allergy	21.49	14.4–32.9
Exposure to AB during pregnancy	1.51	0.96–2.31
BCG-M in the maternity hospital	1.29	1.01–1.67
HepB in the maternity hospital	1.09	0.87–1.37
BCG-M by 12 months of age	0.92	0.66–1.26
HepB by 12 months of age	0.97	0.73–1.28

**Notes:** BCG-M, *M. bovis* BCG-1 (Russia; live attenuated tuberculosis vaccine with reduced antigen content); HepB, hepatitis B vaccine; OR, odds ratio; CI, confidence interval.

**Table 4:** Association between combined BCG and hepatitis B vaccination and atopic dermatitis onset at different age periods.

Vaccination	Age of AD Onset (Months)	Vaccinated n/N (%)	Not Vaccinated n/N (%)	*p*-Value ^1^
Both BCG-M and HepB in the maternity hospital	1–12	199/1446 (13.8)	102/833 (12.2)	0.334
	12–24	81/1446 (5.6)	31/833 (3.7)	0.057
	24–36	21/1446 (1.5)	20/833 (2.4)	0.139
	up to 36	301/1446 (20.8)	153/833(18.4)	0.175
Both BCG-M and HepB by 12 months of age	1–12	263/1956 (13.4)	19/180 (10.6)	0.327
	12–24	105/1956 (5.4)	7/180 (3.9)	0.498
	24–36	35/1956 (1.8)	4/180 (2.2)	0.901
	up to 36	403/1956 (20.6)	30/180 (16.7)	0.246
At least one vaccine in the maternity hospital	up to 36	382/1867 (20.5)	72/412 (17.5)	0.192

**Notes:** ^1^ 2-sample test for equality of proportions with continuity correction. Data are presented as *n*/*N* (%), where *n* is the number of children with AD onset in the specified age period, and *N* is the total number of children in the respective vaccination group.

**Table 5:** Presents the results of these analyses.

AD Onset Period	AB Exposure Period	AD Incidence in AB Group, % (n/N)	AD Incidence in Non-AB Group, % (n/N)	OR	95% CI
12–24 months	1st year of life	6.4% (16/250)	4.7% (96/2029)	1.377	0.797–2.378
24–36 months	2nd year of life	0.9% (3/340)	2.0% (39/1939)	0.434	0.133–1.411

**Notes:** Data are presented as *n*/*N* (%); OR, odds ratio; CI, confidence interval.

## Data Availability

The data presented in this study are available on request from the corresponding author. The data are not publicly available due to privacy and ethical restrictions related to patient medical records.
